# Supplementary material for: The Pathogen Aeromonas salmonicida achromogenes Induces Fast Immune and Microbiota Modifications in Rainbow Trout
Source: Microorganisms. 2023 Feb 20;11(2):539. doi: 10.3390/microorganisms11020539 (PMC9964013; doi:10.3390/microorganisms11020539)
Supplement: Supplementary file 1 [file microorganisms-11-00539-s001.zip › microorganisms-2098126-supplementary.pdf]

microorganisms

MDPI

Article

The pathogen *Aeromonas salmonicida achromogenes* induces fast immune and microbiota modifications in rainbow trout

Baptiste Redivo [1], Nicolas Derôme [2], Patrick Kestemont [1] and Valérie Cornet [1,*]

[1] Research Unit in Environmental and Evolutionary Biology (URBE), Institute of Life, Earth & Environment (ILEE), University of Namur, 5000 Namur, Belgium
[2] Département de Biologie, Institut de Biologie Intégrative et des Systèmes (IBIS), Université Laval, Québec, QC G1V 0A6, Canada
* Correspondence: valerie.cornet@unamur.be

Supplementary materials

Supplementary Materials S1. Mean Ct values of 3 technical replicates obtained by qPCR of several genes expressed in trout head kidney (K) at 0, 6, 24 and 72 hpi.

		Mean Ct values of 3 technical replicates									
Time	Identification	18S	B-actin	mpo	lyso	C3-4	tgfb	il10	vapa	il1b	tnfa
0h	K_0h_1	12,50	21,34	18,56	21,98	31,00	22,30	29,50	34,39	29,68	30,52
0h	K_0h_2	12,55	21,58	19,48	22,08	29,94	22,39	31,00	33,97	29,69	31,11
0h	K_0h_3	12,25	21,73	19,42	22,93	30,92	22,63	28,23	34,92	30,67	30,31
0h	K_0h_4	11,85	20,90	19,27	22,39	30,80	22,57	30,57	34,70	30,40	30,59
6h	K_6h_1	12,81	21,77	19,74	22,65	30,34	22,35	29,28	28,92	29,35	30,36
6h	K_6h_2	12,32	21,49	20,12	22,23	30,68	21,95	29,51	28,68	29,69	31,06
6h	K_6h_3	12,69	21,88	20,08	22,96	29,69	22,42	29,01	28,84	29,24	29,94
6h	K_6h_4	12,85	22,10	20,22	23,04	28,78	22,51	31,58	29,47	29,44	32,41
6h	K_6h_5	12,47	21,67	20,24	22,27	31,91	22,27	29,80	29,45	28,97	31,12
6h	K_6h_6	12,89	22,29	20,43	23,05	31,45	22,50	29,21	30,07	30,17	32,05
24h	K_24h_1	13,00	23,27	21,66	23,64	33,49	23,79	31,43	29,55	30,41	33,08
24h	K_24h_2	12,54	21,58	19,05	21,08	29,87	21,68	30,57	23,05	28,14	30,15
24h	K_24h_3	12,57	21,91	19,74	22,06	30,04	22,70	28,96	29,93	29,12	31,84
24h	K_24h_4	12,73	21,87	19,47	21,45	30,24	22,65	31,20	30,35	29,29	30,50
24h	K_24h_5	12,63	21,79	19,76	21,24	29,28	22,40	32,35	27,30	30,31	32,35
24h	K_24h_6	12,53	21,91	19,85	22,90	30,26	22,65	30,57	29,56	29,86	30,50
72h	K_72h_1	12,55	22,19	19,93	22,21	30,07	23,16	29,38	29,66	28,32	30,42
72h	K_72h_2	12,78	22,60	19,85	22,87	31,81	22,88	28,32	28,78	29,85	31,35
72h	K_72h_3	12,73	22,33	19,68	23,66	30,96	22,67	31,20	30,32	30,44	31,33
72h	K_72h_4	12,64	22,22	19,67	22,52	31,12	22,82	30,31	30,05	28,99	30,93
72h	K_72h_5	12,29	21,14	18,65	20,59	30,07	22,46	28,71	30,27	25,81	30,48
72h	K_72h_6	12,30	22,22	19,92	21,77	30,63	22,50	31,56	31,22	29,59	29,51

Supplementary Materials S2. Pairwise comparisons of the different microbiotas (beta-diversity) using Permanova with Benjamini-Hochberg correction. These Permanova were obtained using Bray-Curtis distance (dissimilarity matrix).

pairs	F.Model	R2	p.value	p.adjusted	sig
Fin.0h vs Skin.0h	10.358585	0.3652716	0.00004	0.0005000	***
Fin.0h vs Fin.6h	1.566467	0.0843708	0.14677	0.1467700	
Fin.0h vs Fin.72h	4.100124	0.2265246	0.00250	0.0057692	**
Fin.0h vs Gills.0h	7.449027	0.3046758	0.00008	0.0005400	***
Fin.0h vs Water.0h	8.130346	0.4249973	0.00354	0.0072000	**
Skin.0h vs Skin.6h	1.974775	0.1098637	0.14220	0.1467700	
Skin.0h vs Gills.0h	20.917466	0.5516578	0.00002	0.0005000	***
Skin.0h vs Water.0h	39.690005	0.7829947	0.00360	0.0072000	**
Skin.0h vs Skin.72h	9.069959	0.3931502	0.00057	0.0019000	**
Water.6h vs Fin.6h	13.189114	0.5687632	0.00498	0.0078632	**
Water.6h vs Skin.6h	46.588421	0.8380958	0.00603	0.0086286	**
Water.6h vs Water.0h	45.152813	0.9186211	0.10000	0.1071429	
Water.6h vs Gills.6h	10.944665	0.5487515	0.00604	0.0086286	**
Water.6h vs Water.72h	6.172906	0.6067987	0.10000	0.1071429	
Fin.6h vs Fin.72h	4.317148	0.2492990	0.00641	0.0087409	**
Fin.6h vs Skin.6h	19.874060	0.5698809	0.00005	0.0005000	***
Fin.6h vs Gills.6h	11.178522	0.4270112	0.00009	0.0005400	***
Fin.72h vs Gills.72h	8.159242	0.4493162	0.00224	0.0057250	**
Fin.72h vs Water.72h	15.809192	0.6931062	0.01162	0.0141240	*
Fin.72h vs Skin.72h	10.850875	0.5204038	0.00226	0.0057250	**
Gills.72h vs Gills.0h	10.869866	0.4553803	0.00023	0.0009857	***
Gills.72h vs Gills.6h	14.281280	0.5434012	0.00031	0.0011625	**
Gills.72h vs Water.72h	31.632594	0.8188058	0.01177	0.0141240	*
Gills.72h vs Skin.72h	24.979485	0.7141181	0.00229	0.0057250	**
Skin.6h vs Gills.6h	16.101487	0.5349067	0.00015	0.0007500	***
Skin.6h vs Skin.72h	8.346270	0.4102113	0.00446	0.0074333	**
Gills.0h vs Water.0h	6.921160	0.4090240	0.00439	0.0074333	**
Gills.0h vs Gills.6h	5.347262	0.2628001	0.00423	0.0074333	**
Water.0h vs Water.72h	49.779669	0.9256225	0.10000	0.1071429	
Water.72h vs Skin.72h	65.716471	0.9037357	0.01170	0.0141240	*

Supplementary Materials S3: Pairwise comparisons of the different microbiotas (beta-diversity) using Permanova with Benjamini-Hochberg correction. These Permanova were obtained using Weighted Unifrac distance.

pairs	F.Model	R2	p.value	p.adjusted	sig
Fin_0h vs Skin_0h	9.9305163	0.3555436	0.0001	0.0007500	***
Fin_0h vs Fin_6h	0.6263704	0.0355360	0.6491	0.6491000	
Fin_0h vs Fin_72h	6.2363156	0.3081745	0.0024	0.0051429	**
Fin_0h vs Gills_0h	13.4897227	0.4424351	0.0003	0.0012857	**
Fin_0h vs Water_0h	11.1362262	0.5030770	0.0037	0.0065294	**
Skin_0h vs Skin_6h	5.2289337	0.2463117	0.0333	0.0384231	*
Skin_0h vs Gills_0h	27.2732772	0.6160212	0.0002	0.0012000	**
Skin_0h vs Water_0h	56.8449344	0.8378656	0.0034	0.0063750	**
Skin_0h vs Skin_72h	12.8292470	0.4781814	0.0001	0.0007500	***
Water_6h vs Fin_6h	15.2702898	0.6042784	0.0045	0.0075000	**
Water_6h vs Skin_6h	70.2698943	0.8864638	0.0057	0.0090000	**
Water_6h vs Water_0h	89.0882374	0.9570300	0.1000	0.1034483	
Water_6h vs Gills_6h	11.7017937	0.5652551	0.0078	0.0117000	*
Water_6h vs Water_72h	24.5996082	0.8601379	0.1000	0.1034483	
Fin_6h vs Fin_72h	7.9795159	0.3803480	0.0023	0.0051429	**
Fin_6h vs Skin_6h	26.5388160	0.6388920	0.0001	0.0007500	***
Fin_6h vs Gills_6h	15.5322927	0.5087169	0.0004	0.0015000	**
Fin_72h vs Gills_72h	11.9047400	0.5434778	0.0022	0.0051429	**
Fin_72h vs Water_72h	11.6622707	0.6249117	0.0135	0.0168750	*
Fin_72h vs Skin_72h	9.8588025	0.4964450	0.0026	0.0052000	**
Gills_72h vs Gills_0h	9.4213331	0.4201950	0.0003	0.0012857	**
Gills_72h vs Gills_6h	21.5927105	0.6427796	0.0008	0.0026667	**
Gills_72h vs Water_72h	33.9004332	0.8288527	0.0099	0.0135000	*
Gills_72h vs Skin_72h	42.9811153	0.8112535	0.0016	0.0043636	**
Skin_6h vs Gills_6h	15.0460699	0.5180071	0.0001	0.0007500	***
Skin_6h vs Skin_72h	14.1412549	0.5409555	0.0009	0.0027000	**
Gills_0h vs Water_0h	6.7629673	0.4034469	0.0086	0.0122857	*
Gills_0h vs Gills_6h	4.9807517	0.2492775	0.0165	0.0198000	*
Water_0h vs Water_72h	77.1953903	0.9507361	0.1000	0.1034483	
Water_72h vs Skin_72h	65.5781663	0.9035523	0.0129	0.0168261	*

Supplementary materials S4: Pairwise comparisons of the different microbiotas (beta-diversity) using Permanova with Benjamini-Hochberg correction. These Permanova were obtained using Unweighted Unifrac distance.

pairs	F.Model	R2	p.value	p.adjusted	sig
Fin_0h vs Skin_0h	2.038372	0.1017234	0.0409	0.0454444	*
Fin_0h vs Fin_6h	3.806033	0.1829293	0.0012	0.0032727	**
Fin_0h vs Fin_72h	3.887796	0.2173435	0.0003	0.0012857	**
Fin_0h vs Gills_0h	3.314471	0.1631581	0.0050	0.0093750	**
Fin_0h vs Water_0h	2.729833	0.1988249	0.0277	0.0319615	*
Skin_0h vs Skin_6h	3.945899	0.1978301	0.0003	0.0012857	**
Skin_0h vs Gills_0h	6.202457	0.2673190	0.0001	0.0010000	***
Skin_0h vs Water_0h	5.176748	0.3200117	0.0039	0.0082000	**
Skin_0h vs Skin_72h	5.468346	0.2808840	0.0003	0.0012857	**
Water_6h vs Fin_6h	4.434158	0.3071989	0.0107	0.0152857	*
Water_6h vs Skin_6h	6.044596	0.4017786	0.0061	0.0096316	**
Water_6h vs Water_0h	16.16550	0.8016415	0.1000	0.1034483	
Water_6h vs Gills_6h	4.479516	0.3323203	0.0057	0.0095000	**
Water_6h vs Water_72h	1.164209	0.2254380	0.5000	0.5000000	
Fin_6h vs Fin_72h	3.531183	0.2136074	0.0026	0.0060000	**
Fin_6h vs Skin_6h	3.271963	0.1790701	0.0056	0.0095000	**
Fin_6h vs Gills_6h	4.647942	0.2365613	0.0001	0.0010000	***
Fin_72h vs Gills_72h	2.975787	0.2293338	0.0041	0.0082000	**
Fin_72h vs Water_72h	9.155362	0.5667073	0.0134	0.0160800	*
Fin_72h vs Skin_72h	2.727101	0.2142751	0.0128	0.0160000	*
Gills_72h vs Gills_0h	9.570329	0.4240226	0.0001	0.0010000	***
Gills_72h vs Gills_6h	8.477811	0.4139999	0.0005	0.0018750	**
Gills_72h vs Water_72h	14.90379	0.6804206	0.0122	0.0159130	*
Gills_72h vs Skin_72h	5.480372	0.3540207	0.0021	0.0052500	**
Skin_6h vs Gills_6h	6.725415	0.3245009	0.0003	0.0012857	**
Skin_6h vs Skin_72h	4.919325	0.2907518	0.0007	0.0021000	**
Gills_0h vs Water_0h	2.935376	0.2269262	0.0077	0.0115500	*
Gills_0h vs Gills_6h	5.307294	0.2613491	0.0006	0.0020000	**
Water_0h vs Water_72h	16.21523	0.8021294	0.1000	0.1034483	
Water_72h vs Skin_72h	13.28458	0.6549104	0.0117	0.0159130	*

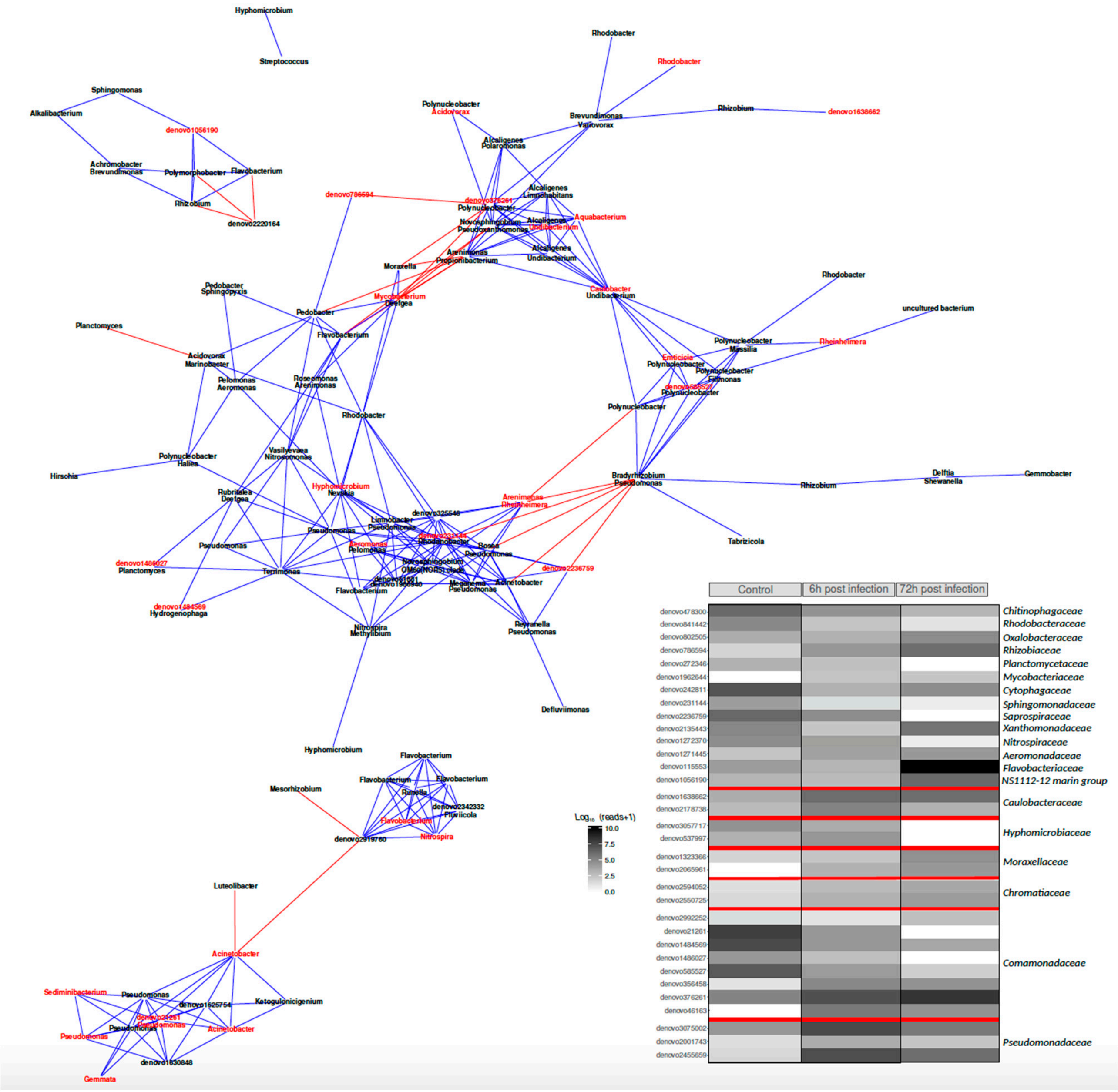

Supplementary Materials S5. Gills correlated network and negative binomial GLM associated on the bottom right. For the network graph, red OTU represent the OTU that are significantly impacted by the bath infection. Blue links represent the positive correlations between OTU's. (r > 0.8) while red ones represent the negative correlations (r < -0.8).

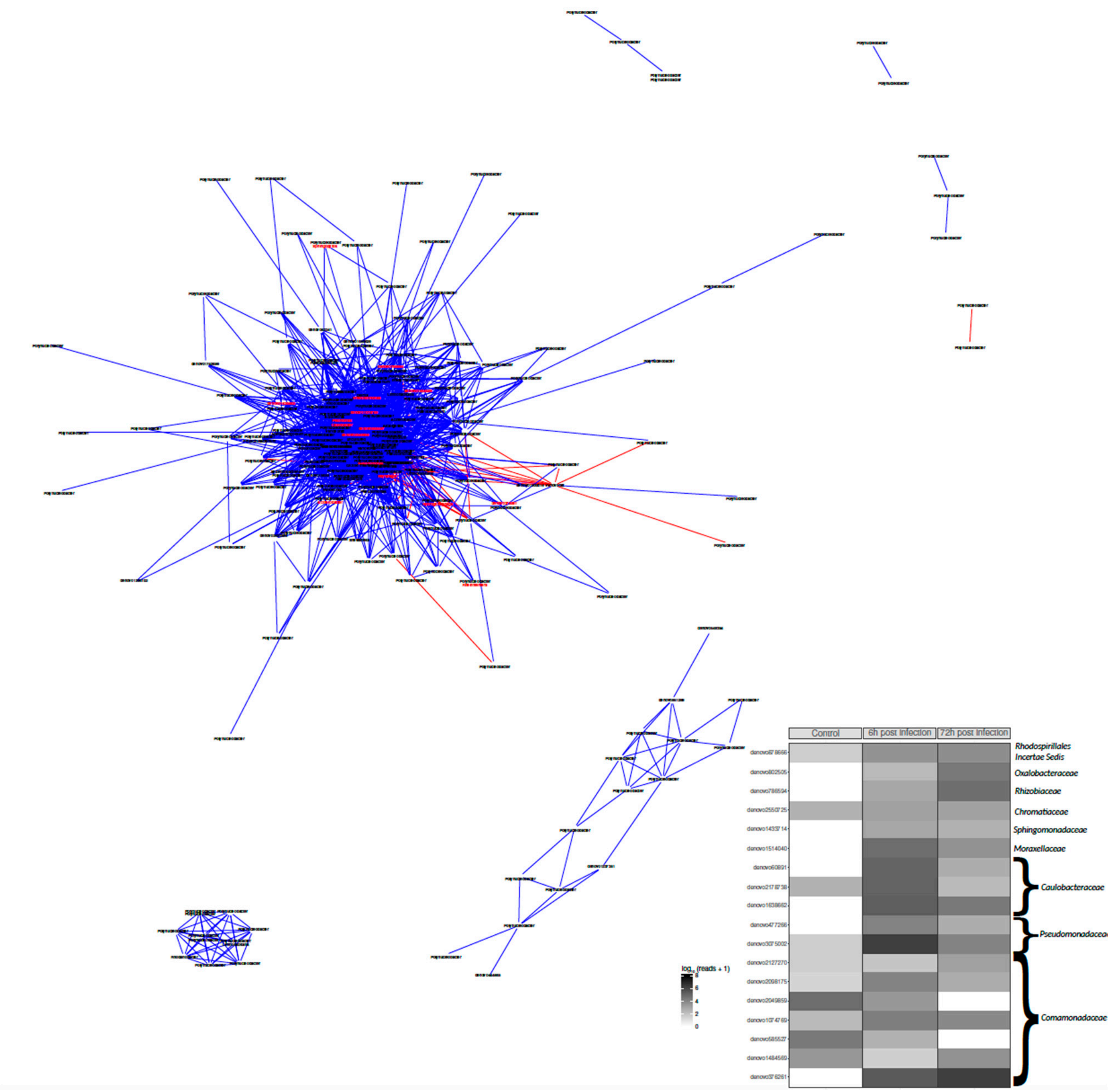

Supplementary Materials S6. Skin correlated network and negative binomial GLM associated on the bottom right. For the network graph, red OTU represent the OTU that are significantly impacted by the bath infection. Blue links represent the positive correlations between OTU's (r > 0.8) while red ones represent the negative correlations (r < -0.8).

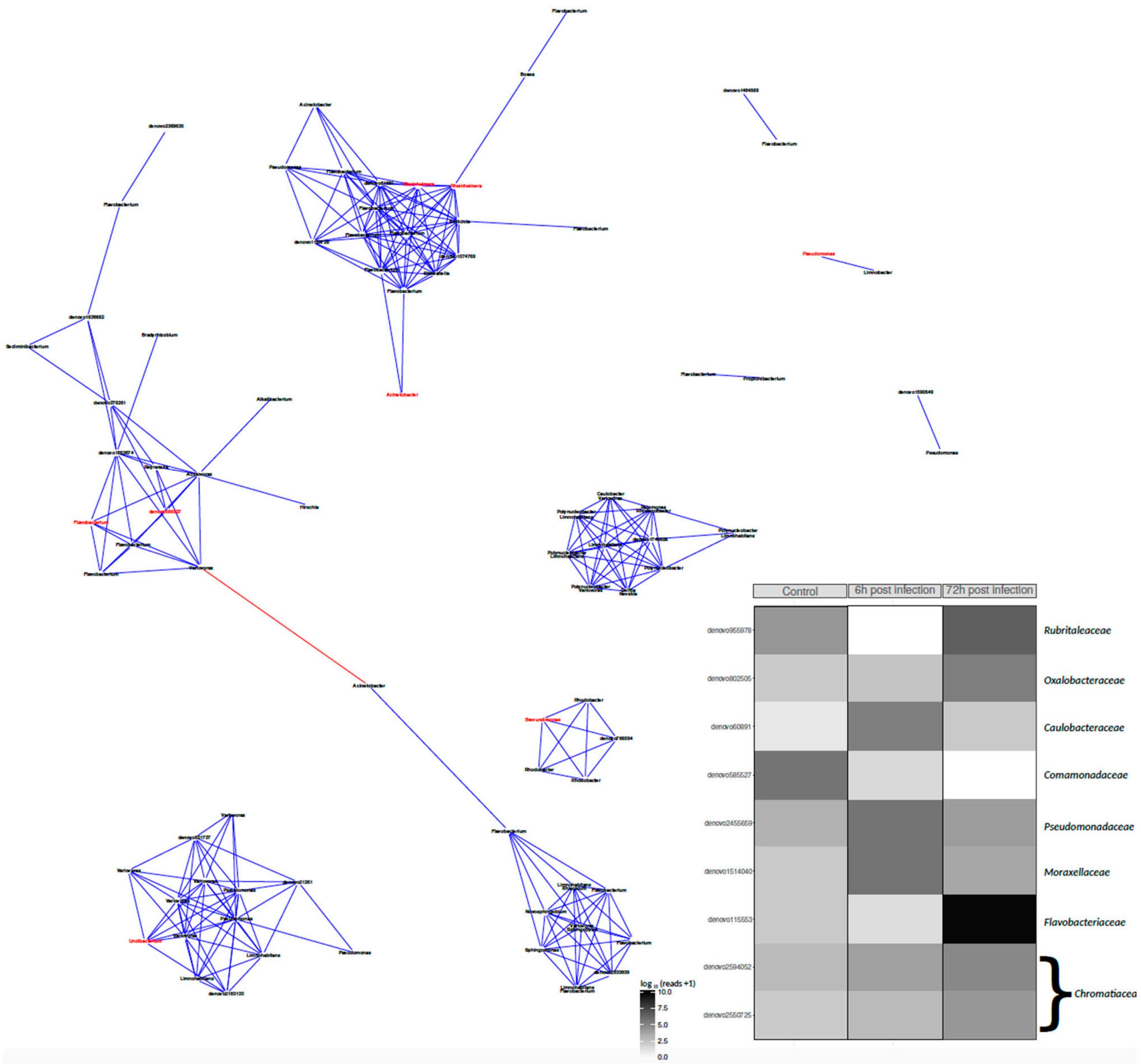

Supplementary Materials S7. Fin correlated network and negative binomial GLM associated on the bottom right. For the network graph, red OTU represent the OTU that are significantly impacted by the bath infection. Blue links represent the positive correlations between OTU's (r > 0.8) while red ones represent the negative correlations (r < -0.8).

Supplementary Materials S8. Pairwise comparisons of the different functional profiles of microbiotas through Tax4Fun using Permanova with Benjamini-Hochberg corrections. These Permanova were obtained using Bray-Curtis distance.

pairs	F.Model	R2	p.value	p.adjusted	sig
Fin.Control vs Fin.6h	0.6387335	0.03621198	0.51143	0.51143	
Fin.Control vs Fin.72h	0.74680378	0.05064174	0.46902	0.4851931	
Fin.6h vs Fin.72h	0.87285857	0.06291844	0.3676	0.39385714	
Skin.Control vs Skin.6h	4.82989152	0.2318731	0.04332	0.06188571	
Skin.Control vs Skin.72h	1.60867633	0.10306296	0.219	0.25269231	
Skin.6h vs Skin.72h	13.0080842	0.52015517	0.00586	0.1276	
Gills.Control vs Gills.6h	6.86167476	0.31386775	0.01351	0.020265	*
Gills.Control vs Gills.72h	11.3843176	0.46687046	0.00045	0.00192857	**
Gills.6h vs Gills.72h	22.4477997	0.65164684	0.00042	0.00192857	**
Water.Control vs Water.6h	64.9053121	0.94194932	0.1	0.12	
Water.Control vs Water.72h	52.1536002	0.92876681	0.1	0.12	
Water.6h vs Water.72h	34.8999517	0.89717211	0.1	0.12	
Fin.Control vs Gills.Control	10.5575615	0.38310942	0.00019	0.001425	**
Fin.Control vs Skin.Control	21.4651701	0.54390162	0.00008	0.0012	**
Fin.Control vs Water.Control	12.5389474	0.53268938	0.00334	0.00949091	**
Skin.Control vs Water.Control	192.387503	0.94591605	0.00348	0.00949091	**
Gills.Control vs Skin.Control	14.3194159	0.45720571	0.0004	0.00192857	**
Gills.Control vs Water.Control	7.12997162	0.41622787	0.00468	0.0117	*
Fin.6h vs Gills.6h	20.3739965	0.5759597	0.00014	0.0014	**
Fin.6h vs Skin.6h	40.4192883	0.72933611	0.00003	0.0009	***
Fin.6h vs Water.6h	3.83279519	0.27708031	0.04986	0.06799091	
Gills.6h vs Skin.6h	4.96227501	0.26169197	0.00747	0.01400625	*
Gills.6h vs Water.6h	12.8436689	0.5879813	0.00598	0.01276	*
Skin.6h vs Water.6h	157.893818	0.9460735	0.00638	0.01276	*
Fin.72h vs Gills.72h	1.2447364	0.11069503	0.28324	0.31471111	
Fin.72h vs Skin.72h	52.8780717	0.84096204	0.00211	0.0072	**
Fin.72h vs Water.72h	13.9288544	0.66553353	0.01265	0.01997368	*
Gills.72h vs Skin.72h	28.6355463	0.741171	0.00216	0.0072	**
Gills.72h vs Water.72h	7.86194649	0.52899844	0.01191	0.01985	*
Skin.72h vs Water.72h	158.637587	0.95773906	0.01166	0.01985	*
